# Ultrasensitive DNA‐Biomacromolecule Sensor for the Detection Application of Clinical Cancer Samples

**DOI:** 10.1002/advs.202102804

**Published:** 2022-01-02

**Authors:** Fengqin Li, Weiqiang Yang, Bingru Zhao, Shuai Yang, Qianyun Tang, Xiaojing Chen, Huili Dai, Peifeng Liu

**Affiliations:** ^1^ State Key Laboratory of Oncogenes and Related Genes Shanghai Cancer Institute RenJi Hospital School of Medicine Shanghai Jiao Tong University Shanghai 200032 China; ^2^ Central Laboratory Renji Hospital School of Medicine Shanghai Jiao Tong University Shanghai 200127 China; ^3^ Micro–Nano Research and Diagnosis Center RenJi Hospital School of Medicine Shanghai Jiao Tong University Shanghai 200127 China; ^4^ Emergency Department RenJi Hospital School of Medicine Shanghai Jiao Tong University Shanghai 200127 China

**Keywords:** aptamers, biomacromolecules detection, clinical cancer samples, electrochemical, luteinizing hormones

## Abstract

Diagnostic testing of biological macromolecules is of great significance for early warning of disease and cancer. Nevertheless, restricted by limited surface area and large steric hindrance, sensitive detection of macromolecules with interface‐based sensing method remains challenging. Here, a “biphasic replacement” electrochemical aptamer‐based (BRE‐AB) sensing strategy which placed capture reaction of the biomacromolecule in a homogeneous solution phase and replaced with a small diameter of single‐stranded DNA to attach to the interface is introduced. Using the BRE‐AB sensor, the ultrasensitive detection of luteinizing hormone (LH) with the detection limit of 10 × 10^−12^ m is demonstrated. Molecular Dynamics simulations are utilized to explore the binding mechanism of aptamer and target LH. Moreover, it is confirmed that the BRE‐AB sensor has excellent sensing performance in whole blood and undiluted plasma. Using the BRE‐AB sensor, the LH concentrations in 40 clinical samples are successfully quantified and it is found that LH is higher expressed in breast cancer patients. Furthermore, the sensor enables simple, low‐cost, and easy to regenerate and reuse, indicating potentially applicable for point‐of‐care biological macromolecules diagnostics.

## Graphical abstract

A biphasic replacement electrochemical aptamer‐based (BRE‐AB) sensing platform is designed and constructed for the ultrasensitive detection of biomacromolecule. This sensor has the advantages of ultrahigh sensitivity, excellent versatility and reusability, and successfully quantifies luteinizing hormone concentrations in cancer samples. The BRE‐AB sensor is an ideal candidate for macromolecular detection and holds great potential for the early diagnosis of cancer.

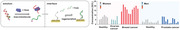

## 1 Introduction

Diagnostic testing of biological macromolecules in vitro, such as hormones and proteins, plays a crucial role for early warning of disease and cancer and is helpful for clinical guidelines in the regulation of organism function to maintain body health.^[^ ^1^, ^2^, ^3^, ^4^ ^]^ Currently, the mainstream approaches for the detection of biological macromolecules depend on western blots^[^ ^5^ ^]^ and enzyme‐linked immunosorbent assays (ELISAs)^[^ ^6^ ^]^ because of their high sensitivity and selectivity. Nonetheless, shortcomings including expensive instruments and reagents, multistep, and difficult to be regenerated also exist. With increasing focus on health concepts, there is an urgent demand to develop cost‐effective, simple, and sensitive detection platforms for more frequent and point‐of‐care diagnostics.^[^ ^7^ ^]^

In recent years, as the continuous advances of selective evolution of ligands by exponential enrichment (SELEX) technology, the electrochemical aptamer‐based (E‐AB) sensors have a great promise to become low‐cost, simple, and sensitive point‐of‐care diagnostic systems.^[^ ^8^, ^9^, ^10^, ^11^, ^12^ ^]^ For example, Plaxco and colleagues developed a E‐AB sensor to directly detect platelet‐derived growth factor in blood serum with a detection limit of 50 × 10^−12^ m.^[^ ^13^ ^]^ Moreover, they demonstrated that E‐AB sensor can continuous and real‐time measure drug pharmacokinetics in living animals.^[^ ^14^ ^]^ Recently, Tanner et al. obtained an aptamer with high affinity binding of luteinizing hormone (LH) by SELEX technology and a robotic E‐AB reader has been established for continuous monitoring of LH pulsatility.^[^ ^15^ ^]^ E‐AB sensors record electrochemical signal generated by the conformational change of the aptamers specifically binding to a molecular target. Although the E‐AB sensors enable to detect various targets, including metal ion, drug molecule and protein, the detection sensitivity for biomacromolecules (>5 nm) is still at the nanomolar level due to limited surface area and large steric hindrance.^[^ ^16^ ^]^ Thus, reducing the steric hindrance at the interface caused by the target macromolecules for sensitive detection has been the focus of research efforts.^[^ ^17^, ^18^ ^]^

**Figure 1:**
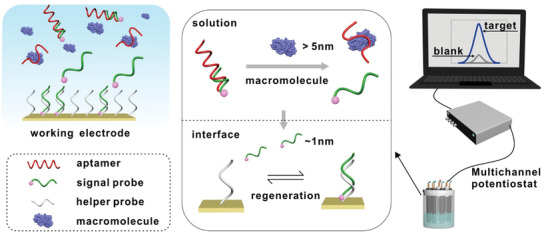
Schematic illustration of the BRE‐AB system.

To overcome this drawback and drive translational applications, herein, we developed a “biphasic replacement” E‐AB (BRE‐AB) sensing platform for ultrasensitive biomacromolecules detection. For this sensor, we placed capture reaction of the biomacromolecule in a homogeneous solution phase and replaced with a small diameter (≈1 nm) of single‐stranded DNA to attach to the interface, thus greatly reducing the steric hindrance of the interface (**Figure** **1**). The BRE‐AB sensor can detect LH with a detection limit of 10 × 10^−12^ m, which is 3 orders of magnitude below the previously reported methods.^[^ ^15^ ^]^ Crucially, molecular dynamics (MD) simulations were utilized to provide structural information on the LH binding aptamer and binding mechanism. Moreover, the BRE‐AB sensor showed excellent sensing performance in complex matrices (whole blood and plasma) and effectively quantify the LH concentrations in 40 clinical patients (6 healthy women, 4 ovarian cancer patients, 10 breast cancer patients, 10 healthy men, and 10 prostate cancer patients). Furthermore, the sensor enables simple, low‐cost, and easy to regenerate and reuse, indicating potentially applicable for point‐of‐care biological macromolecules diagnostics.

## 2 Results and Discussion

### 2.1 Signaling Mechanism

The signaling mechanism of BRE‐AB system was shown in Figure 1. The BRE‐AB system involves a solution reaction and an interface reaction. In the absence of target, prehybridized aptamer/signal duplexes were in the solution phase, and few of free signal probes labeled with redox indicator methylene blue (MB) could enter the interface. Meanwhile, helper probes anchored on the interface were in a steady state. After adding target biomacromolecules, it bound specifically to the aptamer and released signal probes from aptamer/signal duplexes to form more stable aptamer/target complexes. These released signal probes enter the interface and then hybridize with the helper probes, which were anchored on gold electrode surface via Au–S chemistry. Therefore, the MB indicators get close to the gold surface easily, resulting in the accelerated electron transfer greatly.

### 2.2 Experimental and Theoretical Analysis of LH Detection with BRE‐AB

**Figure 2:**
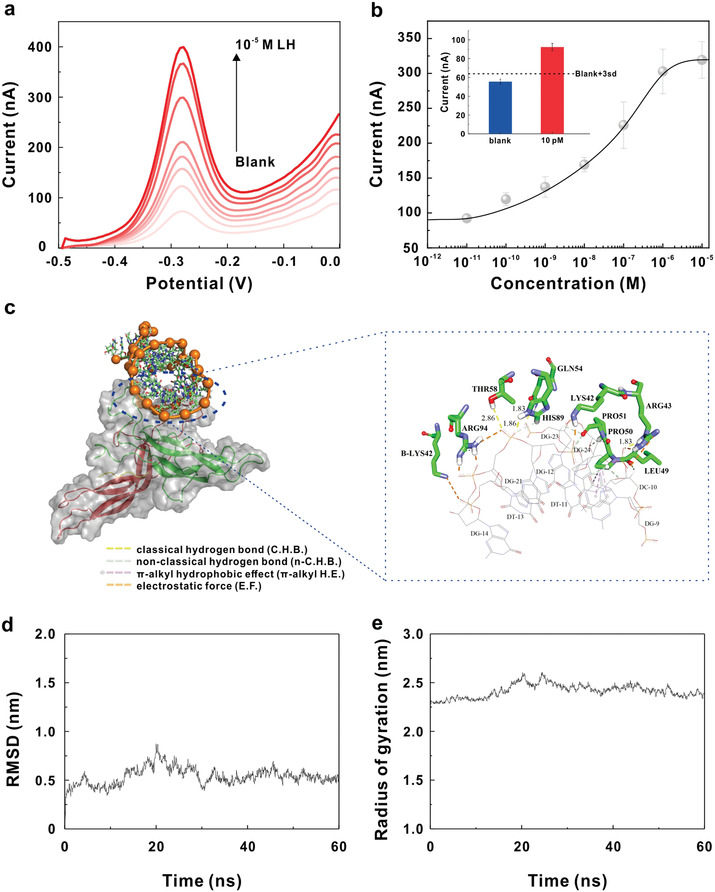
Experimental and theoretical analysis of LH detection with BRE‐AB. a) Square Wave Voltammograms of before and after targets with various concentrations (10^−11^, 10^−10^, 10^−9^, 10^−8^, 10^−7^, 10^−6^, 10^−5^ m). b) Dose‐response curves of LH in phosphate buffer saline (PBS) （10 × 10^−3^ m Na_2_HPO4, 10 × 10^−3^ m NaH_2_PO4, 1 m NaCl, pH 7.4）. The inset figure showed that the current signal of 10 × 10^−12^ m LH was higher than the background signal plus threefold standard deviation and then the detection limit of the BRE‐AB sensor was determined to be 10 × 10^−12^ m. c) Binding mode of aptamer to LH. d) RMSD changing diagram during simulation. e) Radius of gyration changing diagram during simulation.

As a proof‐of‐principle, we first investigated our electrochemical platform for the detection of LH, which is a gonadotrophin produced by the pituitary gland.^[^ ^19^ ^]^ A specific aptamer which can bind to LH with high affinity has been employed in this study (Figure S6, Supporting Information). Before detecting the LH, aptamer bound to signal probe to form an aptamer/signal duplex and the binding delta G was calculated to be 24.52 kcal mol^−1^ using IDT software (Figure S7, Supporting Information). Under optimized conditions, with increasing concentrations of LH from 0 to −10 × 10^−6^ m, we observed enhanced current response (**Figure** **2**a), demonstrating that is concentration‐dependent. These results illustrated that the LH displaced signal probe and bound to aptamer to form a more stable aptamer/LH complex, resulting signal probe released and hybridized with helper probe on the interface, thereby promoting efficiently electron transfer. In addition, the signal collection of the BRE‐AB sensor can be completed within 30 min. Importantly, the detection limit was confirmed to be 10 × 10^−12^ m (Figure 2b), which is 3 orders of magnitude lower than the previously reported method. The improved sensitivity is mainly ascribed to the very small steric hindrance on the interface phase because of using small molecule (signal probe, ≈1 nm) rather than macromolecular (> 5 nm) approached the sensing surface.

The binding of aptamer to LH is the reason that activates the electrochemical response for the sensor. For the displacement‐based sensor in this study, the duplex disruption induced by the binding of aptamer to LH is the key to activate the sensor. Molecular docking and MD simulation method were used to investigate the binding mode of aptamer to LH. The 3D modeling of aptamer showed that there existed a unimolecular chair *G*‐quadruplex structure formed by 24 sequences of GGTATGCTGTGTGGTATGGGGTGG, which was consistent with the prediction of QGRS Mapper software. The formation of *G*‐quadruplex structure enhances the stability of aptamer. A 60 ns dynamic simulation was carried out for the dominant aptamer/LH complex confirmed by molecular docking with the corresponding frame at 50 ns being extracted for the analysis of binding mode. As shown in Figure 2c, LH mainly binded to the surface of aptamer *G*‐quadruplex structure with the residues of B chain. A total of 10 residues of LH and 9 bases of aptamer *G*‐quadruplex structure participated in the binding. The main forces were electrostatic interaction, hydrogen bonding and *π*‐alkyl hydrophobic effect. The detailed bonding type, involved bonding residues or bases, and corresponding atoms are listed in **Table** **1**. The binding energy distribution calculated by a g_mmpbsa method is shown in Table S1 (Supporting Information). As shown from the table, the electrostatic interaction was dominant with the binding free energy of −5217±135 KJ mol^−1^ which was much less than −24.52 kcal mol^−1^ of the duplex. Although the force may be weakened by the formation of duplex, it is strong enough to disrupt the duplex to release the signal probe to activate the sensor. Figure 2d,e shows that after 30 ns, the RMSD fluctuation is less than 2 Å and the radius of gyration is stable after convergence, which validates the effectiveness of simulation process.

**Table 1:** Bonding type formed between LH and its aptamer

Bonding type	Bonding residues/bases involved		Bonding type	Bonding residues/bases involved	
	Residues [atoms]	Bases [atoms]		Residues [atoms]	Bases [atoms]
C.H.B.^a)^	THR58 (HG1)	DT‐13 (O1P)	n‐C.H.B.	PRO51 (O)	DG‐23 (H4’)
C.H.B.	HIS89 (HE2)	DT‐13 (O2P)	n‐C.H.B.	PRO51 (O)	DG‐23 (H1’)
C.H.B.	GLN54 (HE22)	DG‐23 (O1P)	n‐C.H.B.	PRO51 (HD1)	DG‐24 (O4’)
C.H.B.	ARG43 (HH21)	DT‐11 (O1P)	n‐C.H.B.	PRO50 (HD1)	DC‐10 (O4’)
C.H.B.	ARG43 (HE)	DT‐11 (O1P)	*π*‐alkyl H.E.	PRO50 (CG)	DG‐9 (I.R.)
C.H.B.	LYS42 (HZ1)	DG‐12 (O1P)	*π*‐alkyl H.E.	PRO50 (CG)	DC‐10 (P. R.)
C.H.B.	ARG94 (HH12)	DG‐14 (O1P)	*π*‐alkyl H.E.	PRO50 (CG)	DG‐24 (P. R.)
C.H.B.	ARG94 (HH22)	DG‐14 (O2P)	E.F.	LYS42 (NZ)	DG‐21 (O1P)
n‐C.H.B.	HIS89 (HE1)	DT‐13 (O2P)	E.F.	ARG43 (NH1)	DT‐11 (O2P)
n‐C.H.B.	LEU49 (O)	DC‐10 (H4’)	E.F.	ARG94 (NH2)	DT‐13 (O1P)

^a)^ C.H.B. = classical hydrogen bond; n‐C.H.B. = nonclassical hydrogen bond; *π*‐alkyl H.E. = *π*‐alkyl hydrophobic effect; E.F. = electrostatic force; I.R. = imidazole ring; P.R. = pyrimidine ring.

### 2.3 Specificity, Versatility, Reusability, and Storage Time of BRE‐AB

**Figure 3:**
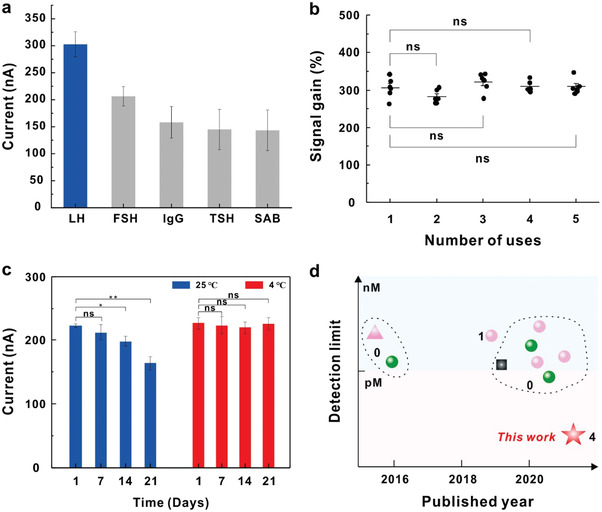
Performance investigation of the proposed BRE‐AB sensor. a) The specificity of the BRE‐AB sensor was investigated when challenged with different proteins at the same concentration (1 × 10^−6^ m FSH, 1 × 10^−6^ m IgG, 1 × 10^−6^ m TSH, 1 × 10^−6^ m SAB, and 1 × 10^−6^ m LH, respectively). b) The signal gain values (%) of the BRE‐AB sensor were calculated over four regeneration and reuse cycles. n.s., nonsignificant. One cycle of the assay takes less than 1 h, including 15 min for system balance, 30 min for signal collection, and 1 min for electrode rinsing. c) Analysis of storage time of the pretreated Au electrodes. d) Comparison of key performances of the BRE‐AB sensors and the previously reported sensors. Numbers in the figure represent the number of regenerations and reuses. The star symbol represents the BRE‐AB sensor in this work. The circles symbols represent aptamer‐based sensors, where pink and green symbols represent electronic and optical detection means, respectively. The pink triangle represents the electrochemical method without using an aptamer and the black square represents a colorimetric method.

In order to evaluate the specificity of the BRE‐AB sensor, we performed control experiments with four interferential proteins, including follicle‐stimulating hormone (FSH), Immune globulin G (IgG), thyroid stimulating hormone (TSH), and serum albumin (SAB). As shown in **Figure** **3**a, the current generated by LH was ≈1.5 folds larger than that of FSH, though they share a similar structure. In addition, 1 × 10^−6^ m of other three proteins caused an apparent decrease in the SWV current response. To be clear, the specificity of the BRE‐AB sensor was no better than other reported methods, possibly due to the reverse correlation between sensitivity and specificity.^[^ ^20^, ^21^, ^22^ ^]^ Next, we replaced the LH aptamer with neutrophil gelatinase‐associated lipocalin (NGAL) aptamer and nucleolin (NCL) aptamer of BRE‐AB sensor to capture NGAL and NCL protein, and achieved signal gain of 200.85% and 357.19%, respectively (Figure S8, Supporting Information). By varying aptamer and corresponding probes, the BRE‐AB sensor can be employed to detect other targets of interest, indicating excellent versatility. We further investigated the regenerability and reusability of the BRE‐AB sensor, which is closely related to the sensing cost and time. We recovered the BRE‐AB sensor by simply rinsing the gold electrode with Milli‐Q water for 60 s to remove the signal probe from the helper/signal duplex. After four reused cycles, the current signal value of the BRE‐AB sensor barely changed (Figure 3b; and Figure S9, Supporting Information), indicating that the BRE‐AB sensor has excellent reusability. The storage time of pretreated Au electrodes were examined. The electrodes were placed at 25 ℃ and 4 ℃, respectively, and were measured current response at day 1, day 7, day 14, and day 21. As shown in Figure 3c, the Au electrodes remained approximately unchanged current signal for 21 days when stored at 4 ℃, while when stored at 25 ℃ gradually decreased over time, indicating that Au electrodes can be stored for at least 21 days at 4 ℃. Furthermore, we speculate that the pretreated Au electrode could be stored for years at 4 ℃.^[^ ^23^, ^24^, ^25^ ^]^ In addition, compared with other previously reported methods, the BRE‐AB sensor possesses combined feature of an ultralow limit of detection (LOD), excellent regenerability and reusability, indicating commercial application potential for biomacromolecule detection (Figure 3d; and Table S2, Supporting Information).

### 2.4 Whole Blood and Clinical Cancer Samples Determination

**Figure 4:**
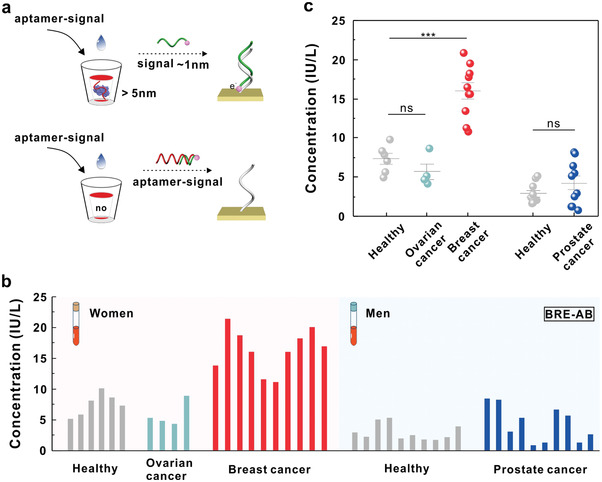
LH detection with BRE‐AB sensor in clinical cancer samples. a) Illustration of BRE‐AB sensor for the detection of biomacromolecule in complex matrices. b) Quantification detection of LH with the BRE‐AB sensor in 40 clinical samples including 6 healthy women, 4 ovarian cancer patients, 10 breast cancer patients, 10 healthy men, and 10 prostate cancer patients. c) Analysis of the LH concentration in clinical samples. ns, nonsignificant; ***P < 0.001 (two‐sample *t* test).

For actual complex matrices, such as plasma and whole blood, the detection sensitivity of a sensor is seriously affected by mass transport, crowding effects, and nonspecific adsorption of environmental components on the sensing interface. Therefore, we then investigated the feasibility of the BRE‐AB sensor in practical complex matrices (**Figure** **4**a). Fortunately, the sensor obtained a LOD in 50% whole blood of 100 × 10^−12^ m (Figures S10 and S11, Supporting Information), suggesting that the BRE‐AB sensor holds great potential application for biomacromolecule detection in clinical samples. Hence, we further investigated the Dose‐response curves and LOD of BRE‐AB sensor in clinical undiluted plasma. As shown in Figures S12 and S13 (Supporting Information), a LOD of 100 × 10^−12^ m was obtained with a dynamic range from 1 × 10^−9^ m to 1 × 10^−12^ m. Inspired by these results and the intent of clinical application, we then utilized the BRE‐AB sensor to quantify LH concentrations in 40 clinical samples, including 6 healthy women, 4 ovarian cancer patients, 10 breast cancer patients, 10 healthy men, and 10 prostate cancer patients (Figure 4b). LH can regulate the synthesis of sex steroid hormones and relate to reproductive diseases. To our knowledge, few studies on the correlation of LH and cancer have been reported. The quantitative results indicated that the BRE‐AB sensor has high concordance with commercial ELISA kit (Figure 4b,c; and Figures S14 and S15, Supporting Information). Moreover, we found that LH is higher expressed in breast cancer patients than healthy women, suggesting that LH may be used as a biomarker in breast cancer (Figure 4c).

## 3 Conclusion

In this work, we have demonstrated a “biphasic replacement” E‐AB (BRE‐AB) sensing platform for highly sensitive detection of biomacromolecules at picomolar level. This sensor was prepared by a simple and low‐cost way, and has the advantages of ultrahigh sensitivity, excellent regenerability and reusability as well. Moreover, even in whole blood, the BRE‐AB sensor also exhibits a detection limit of 100 × 10^−12^ m. The MD simulation results revealed that the main forces of LH binding to aptamer were electrostatic interaction, hydrogen bonding and *π*‐alkyl hydrophobic effect. Guided by theoretical simulation and free energy prediction to design probe sequence reasonably, we speculate that the BRE‐AB sensor can be used to analyze and detect target of interest. This work demonstrates that the BRE‐AB sensor is an ideal candidate for macromolecular detection and holds great potential for the early diagnosis of cancer.

## 4 Experimental Section

### Materials and Reagents

All DNA oligonucleotides were synthesized and purified by Sangon Biotechnology Co., Ltd. (Shanghai, China). Luteinizing hormone (LH), follicle‐stimulating hormone (FSH), and thyroid stimulating hormone (TSH) were obtained from ANPEL Laboratory Technologies Inc. (Shanghai China). Immune globulin G (IgG) and serum albumin (SAB) were purchased from Sigma‐Aldrich Co., Ltd. (USA). Neutrophil gelatinase‐associated lipocalin (NGAL) and nucleolin (NCL) were obtained from Sino Biological (Beijing, China) and Shanghai kanglang Biotech Co., Ltd, respectively. Tris‐(2‐carboxyethyl) phosphine hydrochloride (TCEP) and 6‐Mercaptohexanol (MCH) were also acquired from Sigma‐Aldrich Co., Ltd. (USA). Human LH ELISA Kit was also purchased from Sangon Biotechnology Co., Ltd. (Shanghai, China). All of the clinical samples were provided by Renji Hospital (Shanghai, China). Rabbit whole blood was obtained from Ruite Biotechnology Co., Ltd. (Guangzhou, China). The PBS buffer involved used was phosphate buffer (10 × 10^−3^ m, pH 7.4) containing 1 m NaCl. The TM buffer used was Tris buffer (20 × 10^−3^ m, pH 8.0) containing 50 × 10^−3^ m MgCl_2_. Deionized water purified by a Millipore system (18.2 MΩ cm) was used throughout the experiment.The DNA sequence used as follow (5’‐3’)

Aptamer: TATGGTATGCTGTGTGGTATGGGGTGGCGTGCTCT
Signal probe: ACACAGCATACCATA‐MB
Helper probe: SH‐(CH_2_)_6_‐TATGGTATGCTGTGT

### Apparatus and Measurements

All the electrochemical experiments were investigated on a CHI 1040C multichannel potentiostat (CH Instruments Co., Ltd., Shanghai, China) at room temperature. A three‐electrode system including a gold working electrode (the diameter was 2 mm), an Ag/AgCl reference electrode and a platinum wire counter electrode was used and the binding reactions interrogation was performed in a custom cell (XIANREN instrument Co., Ltd., Shanghai, China). The square wave voltammetry (SWV) signals were carried out with the potential ranging from −0.5 to 0 V with the amplitude of 50 mV under the frequency of 50 Hz.

### Electrode Preparation

Au electrode (Φ = 2 mm) as the working electrode needs surface pretreatment before sensor fabrication. First, the electrode was polished with a 0.05 µm alumina powder, and ultrasonically rinsed with ethanol and Milli‐Q water for 5 min respectively. Then it was electrochemical cleaned and activated in 0.05 and 0.5 m H_2_SO_4_, respectively. Finally, the electrode was dried under nitrogen stream for further use.

### Sensor Fabrication

The sensor consists of a solution phase and an interface phase. To fabricate the interface phase, the Au electrodes were dropped and incubated with reduced helper probe overnight at room temperature and thus helper probes were immobilized on Au electrodes via Au—S bond. The modified electrodes were then rinsed with Milli‐Q water and further passivated with 2 × 10^−3^ m MCH for 2 h to displace nonspecifically bound oligonucleotides. Meanwhile, the aptamer/signal duplex was prepared that aptamer and signal probe labeled with MB were mixed in TM buffer (20 × 10^−3^ m Tris, 50 × 10^−3^ m MgCl_2_, pH 8.0) and heated to 85 ℃ for 10 min, then cooled to 25 ℃ for next assay. Subsequently, the modified gold working electrode, Ag/AgCl reference electrode and platinum wire counter electrode were placed in a custom cell and immersed in PBS buffer. Before measurement, 60 µL of aptamer/signal duplex was added to 1940 µL of PBS buffer as solution phase for 15 min at room temperature to achieve reaction equilibrium between interface and solution phase.

### Optimization of Experimental Conditions

To achieve optimal detection performance of the BRE‐AB sensor, several parameters involved the sensor fabrication or the electrochemical testing were optimized, including incubation concentration of helper probe, ionic strength of PBS buffer, the concentration ratio between aptamer and signal to form duplexes, the concentration of duplexes in solution phase, and scanning frequency of SWV (**Table** **2**). The corresponding results were illustrated in (Figures S1–S5, Supporting Information). 0.75 × 10^−6^ m was selected as the optimal incubation concentration of the helper probe. The best concentration ratio between aptamer and signal to form aptamer/signal duplexes was 1:1, and the optimal concentration of duplexes in solution phase was 30 × 10^−9^ m. A PBS buffer with 1 m NaCl was chosen as the detection solution and 50 Hz was chosen as the scanning frequency.

**Table 2:** The optimized fabrication and testing parameters for the BRE‐AB sensor

Parameter^a)^	Tested range	Selected value
HP conc. [× 10^−6^ m ]	0.1, 0.25, 0.5, 0.75, 1.0, 1.5	0.75
NaCl conc. [m]	0.1, 0.3, 0.5, 0.7, 1.0, 1.3, 1.5	1.0
Ratio/Apt:SP	1:4, 1:2, 3:4, 1:1, 3:2, 2:1	1:1
Apt/SP duplex conc. [× 10^−9^ m]	10, 20, 30, 40	30
SWV frequency	50, 100, 150, 200	50

^a)^ HP = helper probe; Apt = aptamer; SP = signal probe.

## Conflict of Interest

The authors declare no conflict of interest.

## Supporting information

Supporting Information

## Data Availability

Research data are not shared.
