# Supplementary material for: Ultrasensitive DNA‐Biomacromolecule Sensor for the Detection Application of Clinical Cancer Samples
Source: Adv Sci (Weinh). 2022 Jan 2;9(6):2102804. doi: 10.1002/advs.202102804 (PMC8867190; doi:10.1002/advs.202102804)
Supplement: Supplementary file 1 — Supporting Information [file ADVS-9-2102804-s001.pdf]

ADVANCED SCIENCE
Open Access

Supporting Information

for *Adv. Sci.*, DOI: 10.1002/advs.202102804

Ultrasensitive DNA-biomacromolecule sensor for the detection application of clinical cancer samples

*Fengqin Li, Weiqiang Yang, Bingru Zhao, Shuai Yang, Qianyun Tang, Xiaojing Chen, Huili Dai, Peifeng Liu**

Supporting information

Ultrasensitive DNA-biomacromolecule sensor for the detection application of clinical cancer samples

*Fengqin Li[#], Weiqiang Yang[#], Bingru Zhao[#], Shuai Yang, Qianyun Tang, Xiaojing Chen, Huili Dai, Peifeng Liu**

Supporting information

Ultrasensitive DNA-biomacromolecule sensor for the detection application of clinical cancer samples

*Fengqin Li[#], Weiqiang Yang[#], Bingru Zhao[#], Shuai Yang, Qianyun Tang, Xiaojing Chen, Huili Dai, Peifeng Liu**

Dr. F. Li, B. Zhao, Dr. S. Yang, Dr. Q. Tang, X. Chen, Prof. H. Dai, Prof. P. Liu

State Key Laboratory of Oncogenes and Related Genes, Shanghai Cancer Institute, RenJi Hospital, School of Medicine, Shanghai Jiao Tong University, Shanghai 200032, China

E-mail: lpf@sjtu.edu.cn

Dr. F. Li, B. Zhao, Dr. S. Yang, Dr. Q. Tang, X. Chen, Prof. H. Dai, Prof. P. Liu

Central Laboratory, Renji Hospital, School of Medicine, Shanghai Jiao Tong University, Shanghai 200127, China

Dr. F. Li, B. Zhao, Dr. S. Yang, Dr. Q. Tang, X. Chen, Pro. H. Dai, Prof. P. Liu

Micro–Nano Research and Diagnosis Center, RenJi Hospital, School of Medicine, Shanghai Jiao Tong University, Shanghai 200127, China

W. Yang

Emergency Department, RenJi Hospital, School of Medicine, Shanghai Jiao Tong University, Shanghai 200127, China

[#] *Fengqin Li, Weiqiang Yang and Bingru Zhao contributed equally to this work.*

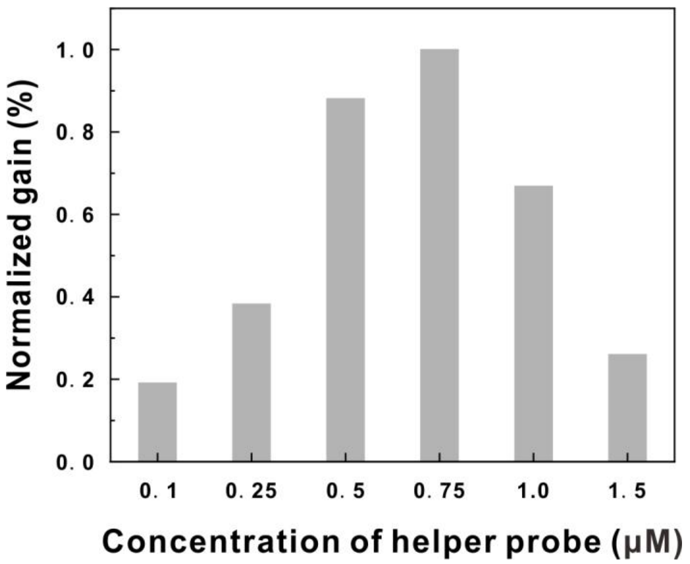

Figure S1. To obtain the maximum signal gain, we optimized the assembly concentration of the helper probe at the interface. Since both high and low density of the helper probe on the electrode surface were unfavorable, so 0.75 μM was chosen as the optimal incubation concentration to realize medium density. We defined signal gain as:

$$Signal\ Gain\ (\%) = {(I - I_0)}/{I_0} \qquad \text{Equation 1}$$

Where, I is the peak current obtained in the presence of the target, and I_0 is the peak current obtained in the blank solution.

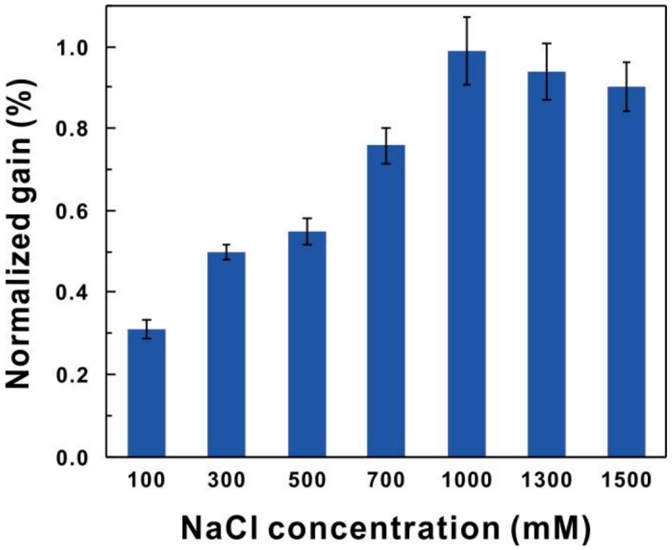

Figure S2. The ionic strength in the PBS buffer solution was optimized. When the concentration of NaCl was 1000 mM, the maximum signal gain was achieved, so 1000 mM was confirmed to be the optimal concentration. Data presented are means ± S.D. from 3 independent experiments.

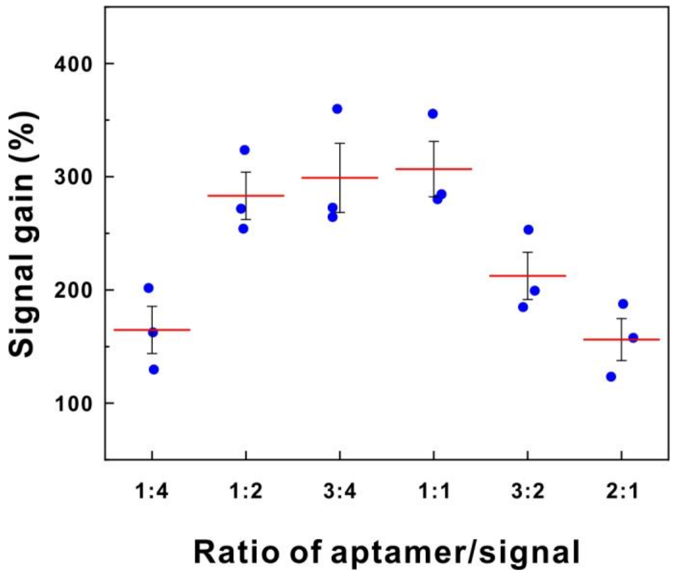

Figure S3. To improve the signal gain of the BRE-AB sensor, we have optimized the concentration ratio between the aptamer and signal probe. When the ratio was 1:1, the signal gain was largest. Therefore, 1:1 was confirmed to be the optimal ratio to synthesize the aptamer/signal duplexes.

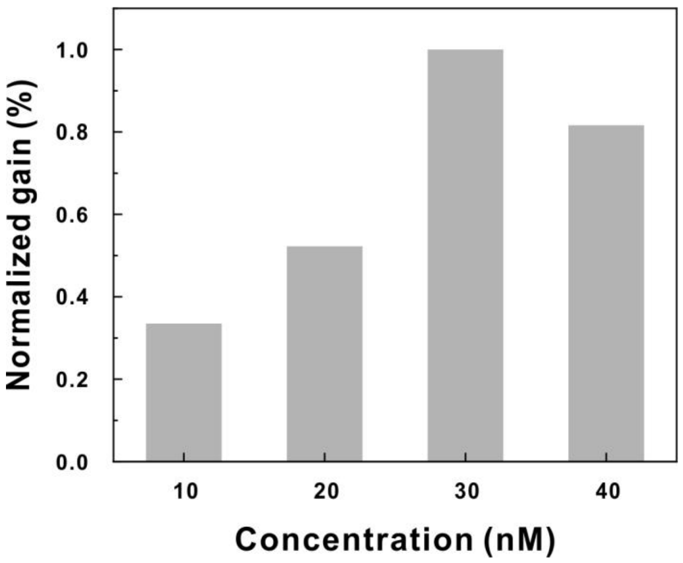

Figure S4. Signal gain at different concentration of aptamer/signal duplex in PBS buffer.

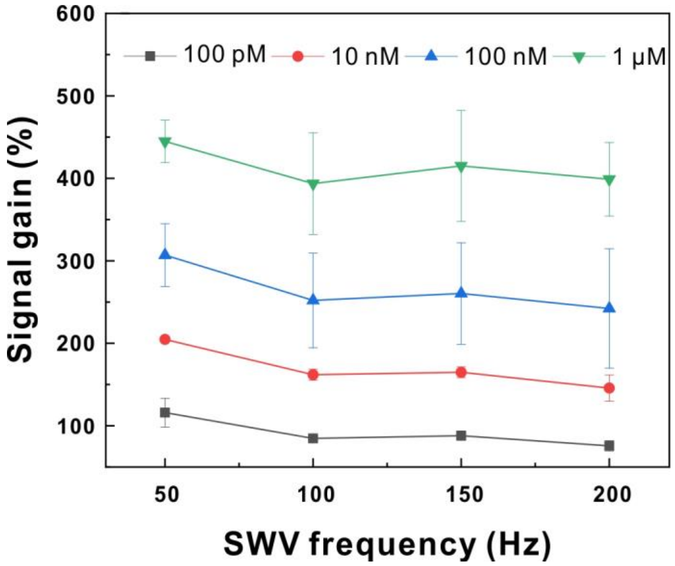

Figure S5. Signal gain at different SWV frequencies. Since frequency is a key influence parameter for the sensor, we applied four concentrations (100 pM, 10 nM, 100 nM and 1 μM) of LH to investigate the signal gain under different SWV frequencies. The results indicated that 50 Hz presented the maximum signal gain at four concentrations and was selected as the test frequency. Considering scan time and current value, lower frequencies were ignored.

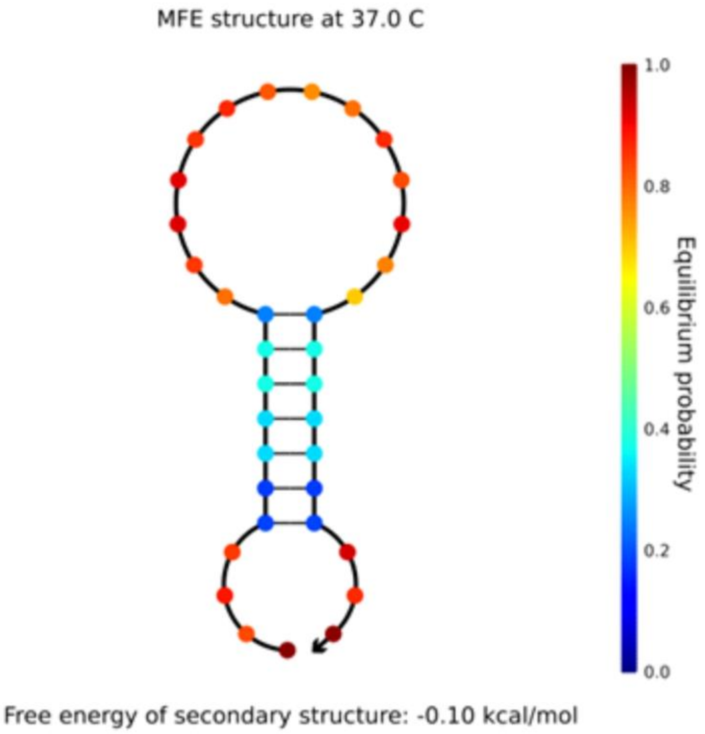

Figure S6. The Gibbs free energy of secondary structure of aptamer was estimated to be -0.10 kcal/mol by NUPACK software. (Web for reference: http://www.nupack.org/).

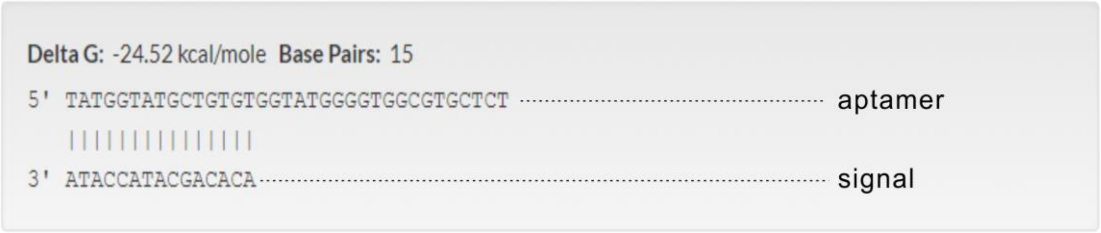

Figure S7. The delta G of aptamer binding to signal probe was calculated to be -24.52 kcal/mol by IDT software. (Web for reference: http://sg.idtdna.com/pages).

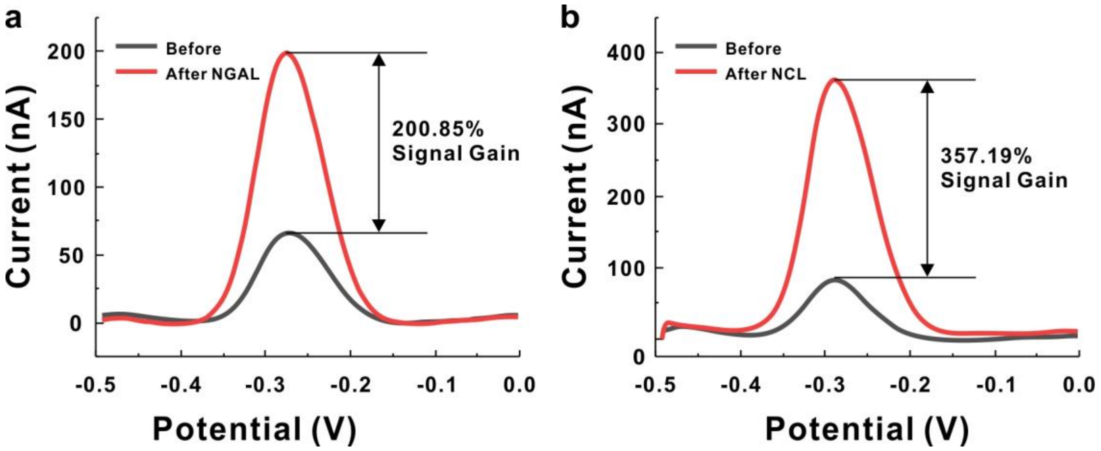

Figure S8. The SWV current responses of the BRE-AB sensor before and after NGAL (a) and NCL (b). The concentration of NGAL and NCL was 100 nM.

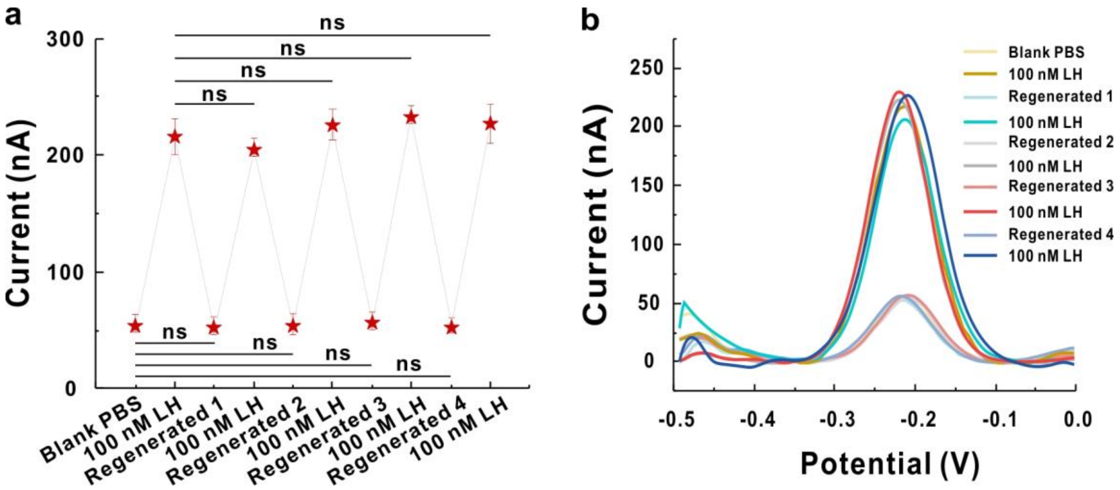

Figure S9. a) The regenerability and reusability of the BRE-AB sensor were verified for four cycles. Data are presented as mean ± s.d., n = 6 independent experiments. ns, nonsignificant. (Student's t-test). b) Four cycles of SWV voltammograms obtained before, after adding 100 nM LH and after regeneration.

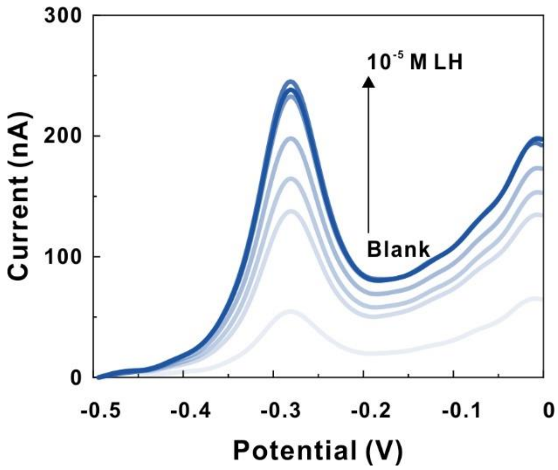

Figure S10. SWV voltammograms obtained before and after adding 10^{-10}, 10^{-9}, 10^{-8}, 10^{-7}, 10^{-6}, 10^{-5} M LH in 50% whole blood.

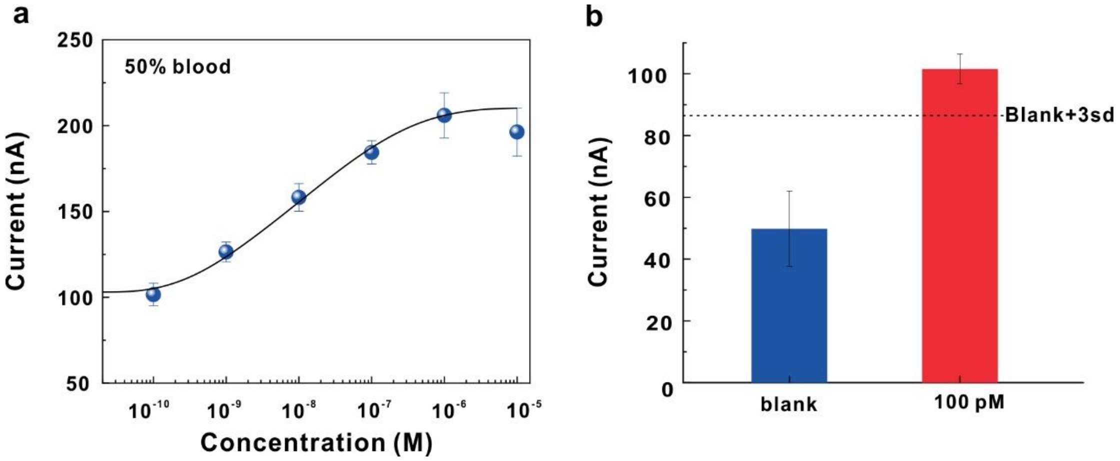

Figure S11. a) Dose-response curves of LH in 50% whole blood. b) The current signal of 100 pM was higher than the background signal plus 3-fold standard deviation and then the detection limit of the BRE-AB sensor was determined to be 100 pM in 50% whole blood.

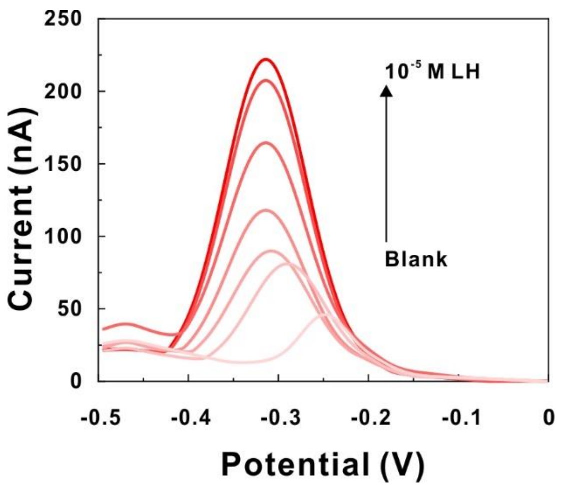

Figure S12. SWV voltammograms obtained before and after adding 10^{-10}, 10^{-9}, 10^{-8}, 10^{-7}, 10^{-6}, 10^{-5} M LH in undiluted plasma.

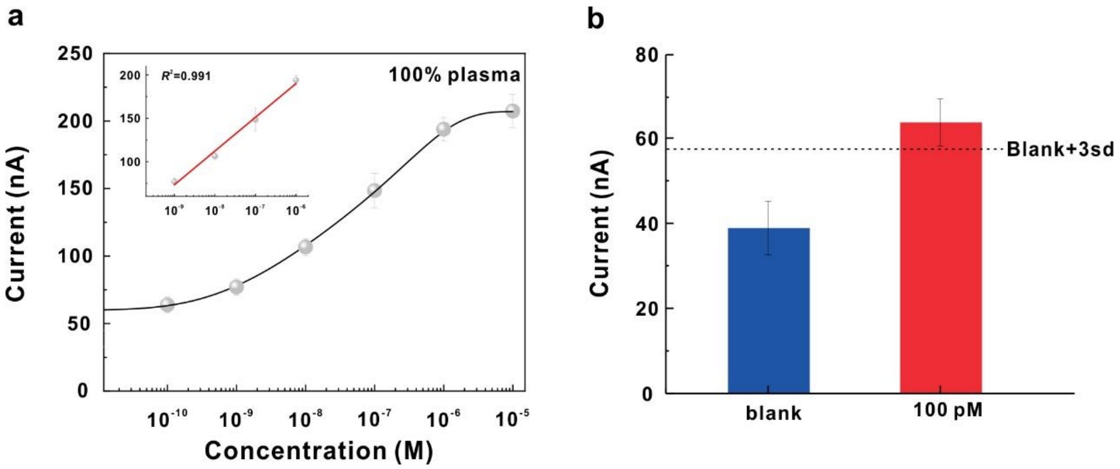

Figure S13. a) Dose-response curves of LH in undiluted plasma. The inset figure showed that the dynamic range was from 1 nM to 1 μM. b) The current signal of 100 pM was higher than the background signal plus 3-fold standard deviation and then the detection limit of the BRE-AB sensor was determined to be 100 pM in undiluted plasma.

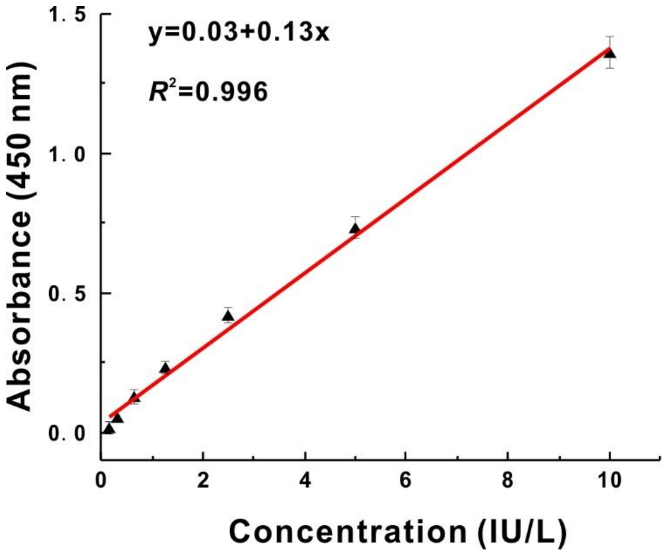

Figure S14. The standard curve of ELISA method was drawn from the absorbance of the standard LH working solution, and the LH concentrations were as follows: 10, 5, 2.5, 1.25, 0.63, 0.31, 0.16, 0 IU/L.

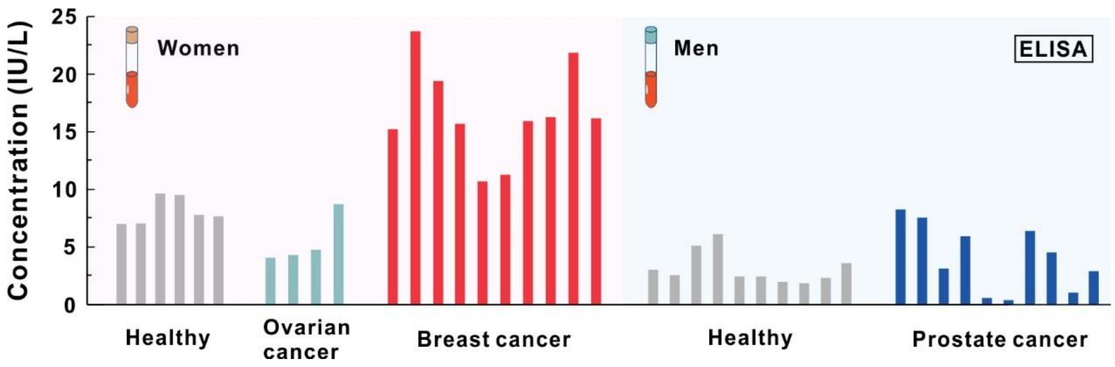

Figure S15. LH detection with ELISA method in clinical samples.

Table S1. Energy type distribution of binding

Energy type	In H_2O (0.1 M NaCl)
	KJ/mol
van der Waal energy	-241±20
Electrostatic energy	-6069±153
Polar solvation energy	1118±87
SASA energy	-26±2
SAV energy	0
WCA energy	0
Binding energy	-5217±135

Table S2. Comparison of macromolecular detection methods.

Detection technology	Aptamer-based	Detection limit	regenerability and reusability	Target type	Reference
Optical spectroscopy	yes	6.7 nM	N/A	Tau protein	Anal. Bioanal. Chem. 2020, 412, 1193-1201
Electrochemical	no	10 nM	N/A	Antibody and streptavidin	J. Am. Chem. Soc. 2015, 137, 15596-15599
Electrochemical	yes	25 nM	N/A	Ranibizumab	Sens. Actuators, B 2020, 312, 127941
Microcantilever	yes	1 nM	N/A	Nucleolin	Talanta 2016, 146, 727-731
Optical spectroscopy	yes	0.2 nM	N/A	Lipocalin 1	Biosens. Bioelectron. 2020, 169, 112607
Electrochemistry	yes	3 nM	N/A	Insulin	Biosens. Bioelectron. 2021, 180, 113124
Electrochemistry	yes	10.9 nM	1	LH	Nat. Commun. 2019, 10, 852
Colorimetry	no	1.5 nM	N/A	Metallothionein	Anal. Chem. 2020, 92, 2080-2087
Field effect transistors	yes	1.75 nM	N/A	HPV-16 E7 protein	Anal. Bioanal. Chem. 2021, 413, 779-787
Electrochemistry	yes	10 pM	4	LH	***This work***

Table S3. Probe sequences used in the study.

Probe name	Sequence (5'-3')
NGAL aptamer	AGCAGCACAGAGGTCAGATGGCGCTGGATAGCAAGATCACGT TATCATCGTAAACCCTATGCGTGCTACCGTGAA
NGAL-SP	CATCCCATCTGAGAC-MB
NGAL-HP	SH-C_6-AGGTCAGATGGCGCT
Nucleolin aptamer	GGTGGTGGTGGTTGTGGTGGTGGTGGTTTTTT
NCL-SP	TTCAACCACCGTATG-MB
NCL-HP	SH-C_6-GTGGTGGTGGTTGTG

MD simulation method

Molecular docking: The Autodocktools software[1] was used to obtain the proper aptamer/LH complex to start MDs with. Semi-flexible docking mode was confirmed using LH as the receptor and the aptamer as the ligand. For achieving a reasonable docking, a crystal structure of the beta subunit of LH (6p57) with PDB format was download from the protein database (https://www.rcsb.org/structure/6P57). Before docking, this 3D-structure complex was processed to remove H_2O and small molecules with PyMol. The 3D-structure modeling of aptamer was carried out using RNAfold and RNAComposer software and then made an energy minimization step using GROMACS software.[2-4] QGRS Mapper software was used to predict if there may form a *G*-quadruplex structure.[5] Molecular dynamics simulation: GROMACS 2019.6 package with an Amber force field of FF99SB-ILDN was used for a systematical simulation. Five steps were mainly involved. 1. The proper aptamer/LH complex was solvated with a SPC216 H_2O model using a cubic box with 10 Å of aptamer to the box wall. 2. The GMX commands, genion and -conc, were repeatedly applied to acquire a simulation system of H_2O containing 0.1 M NaCl. The system was kept neutral with using appropriate number of Na^+ to replace H_2O molecules. 3. A steepest descent algorithm of 50000 steps was carried out for optimizing simulation environment. 4. Both 300 K and 1 bar were set for the constant temperature and pressure, respectively. 5. 50 ns was performed for a complete simulation. MD simulation analysis: Appropriate time frames were extracted for an analysis of the interaction of aptamer to LH using Discovery studio and PyMOL. A g mmpbsa method was used to calculate the binding free energy between the aptamer and LH.[6]

References

[1] G. M. Morris, R. Huey, W. Lindstrom, M. F. Sanner, R. K. Belew, D. S. Goodsell, A. J. Olson, J. Comput. Chem. **2009**, *30*, 2785.

[2] M. Zuker, Nucleic Acids Res. **2003**, *31*, 3406.

[3] M. Antczak, M. Popenda, T. Zok, J. Sarzynska, T. Ratajczak, K. Tomczyk, R. W. Adamiak, M. Szachniuk, Acta Biochim. Pol. **2016**, *63*, 737.

[4] D. Van Der Spoel, E. Lindahl, B. Hess, G. Groenhof, A. E. Mark, H. J. Berendsen, J. Comput. Chem. **2005**, *26*, 1701.

[5] O. Kikin, L. D'Antonio, P. S. Bagga, Nucleic Acids Res. **2006**, *34*, W676.

[6] R. Kumari, R. Kumar, C. Open Source Drug Discovery, A. Lynn, J. Chem. Inf. Model. **2014**, *54*, 1951.
